# Matrine Suppresses Lung Cancer Progression via Dual Inhibition of CHEK1‐Mediated DNA Damage Repair and PI3K/AKT Survival Signaling

**DOI:** 10.1111/1759-7714.70393

**Published:** 2026-09-22

**Authors:** Jianghao Yu, Siyang Chen, Tingting Hou, Xianwu Weng, Yakun Liu, Junchao Huang, Zeshan Yang, Jinyu Bao, Changshuai Zhou, Wanqing Lv, Qingzhe Wu, Yun Xu, Jinshan Zhou

**Affiliations:** ^1^ Department of Cardiothoracic Surgery, Center for Oncology Medical, The Fourth Affiliated Hospital of School of Medicine, and International School of Medicine, International Institutes of Medicine Zhejiang University Yiwu China; ^2^ Department of Respiratory and Critical Care Medicine, Center for Oncology Medicine, the Fourth Affiliated Hospital of School of Medicine, and International School of Medicine, International Institutes of Medicine Zhejiang University Yiwu China; ^3^ Zhejiang Key Laboratory of Precision Diagnosis and Treatment for Lung Cancer Yiwu China

**Keywords:** apoptosis, CHEK1, DNA damage response, dual‐mechanism therapy, lung cancer, matrine, PI3K/AKT pathway

## Abstract

Lung cancer remains a leading cause of cancer‐related mortality worldwide, necessitating the development of novel therapeutic strategies. Matrine, a natural alkaloid derived from *Sophora flavescens* , has demonstrated broad antitumor properties, yet its underlying mechanisms, particularly concerning DNA damage response, remain incompletely elucidated. This study integrates network pharmacology, bioinformatics, and experimental validation to uncover a novel dual‐targeting mechanism of matrine in lung cancer. Bioinformatics analysis identified CHEK1 as a key potential target. In vitro, matrine selectively inhibited proliferation, migration, invasion, and clonogenic survival of A549 and LLC cells, while reducing the G2/M phase proportion, indicating G2/M checkpoint impairment. Mechanistically, matrine concurrently downregulated CHEK1, p‐PI3K, p‐AKT, BCL‐2, and EMT markers (Vimentin, MMP2, MMP9), and upregulated γH2AX, E‐cadherin, Bax, and Cleaved Caspase‐3. Crucially, siRNA experiments revealed that PI3K knockdown induced apoptosis without affecting CHEK1/γH2AX, whereas CHEK1 silencing promoted apoptosis independently of PI3K/AKT, demonstrating complementary. In vivo, matrine significantly inhibited LLC tumor growth in a xenograft model, suppressed proliferation (Ki67), and modulated the CHEK1‐PI3K/AKT axis, corroborated by IHC and Western blotting. Importantly, matrine exhibited no significant systemic toxicity. Our findings unveil that matrine exerts potent antitumor effects by simultaneously inhibiting CHEK1‐mediated DNA repair and PI3K/AKT survival signaling, presenting a novel dual‐targeting approach for lung cancer treatment.

## Graphical abstract

Matrine simultaneously targets CHEK1 (impairing DNA repair, increasing γH2AX) and PI3K/AKT (activating mitochondrial apoptosis), reduced EMT, and strong in vitro and in vivo antitumor effects with low toxicity, positioning it as a promising dual‐mechanism agent for lung cancer.

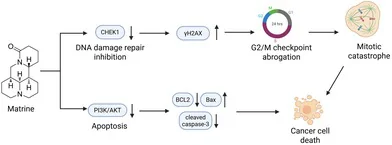

## 1 Introduction

Lung cancer persists as a leading contributor to global cancer‐related mortality, with its high incidence, therapeutic resistance, and metastatic potential posing persistent challenges in clinical management [1, 2, 3]. Conventional chemotherapeutic agents suffer from limitations including poor selectivity and significant toxicity, driving the exploration of natural compounds as promising antitumor therapeutics [4, 5]. Matrine, a principal bioactive alkaloid derived from the leguminous plant *Sophora flavescens* , has demonstrated remarkable antineoplastic properties through multifaceted pharmacological actions [6, 7, 8]. This compound exerts potent antiproliferative and pro‐apoptotic effects in cancer through modulation of the PI3K/AKT signaling axis [9, 10]. In A549 lung adenocarcinoma cells, matrine dose‐dependently suppresses PI3K/AKT pathway phosphorylation while concurrently upregulating the pro‐apoptotic protein Bax and downregulating the anti‐apoptotic protein Bcl‐2, culminating in significantly enhanced apoptosis rates [11, 12]. Beyond apoptosis induction, matrine effectively inhibits epithelial‐mesenchymal transition (EMT) via the PTEN/PI3K/AKT cascade, evidenced by elevated E‐cadherin expression and reduced vimentin levels, thereby suppressing cancer cell migration and invasion [13]. Further pharmacological investigations reveal matrine's capacity to disrupt mitochondrial membrane potential, activate caspase cascades, inhibit topoisomerase II activity, and suppress cancer stemness markers including CD133 and ALDH1A1, collectively highlighting its multi‐target therapeutic potential against lung cancer progression [14].

CHEK1 (Checkpoint kinase 1), a central regulator of the DNA damage response (DDR), assumes a complex role in tumorigenesis [15, 16, 17]. In lung cancer, CHEK1 promotes malignant cell survival by maintaining genomic integrity and orchestrating cell cycle checkpoints [18]. Upon DNA damage, activated CHEK1 induces G2/M phase arrest, creating a critical repair window that enables tumor cells to evade apoptosis [19, 20]. Notably, CHEK1 overexpression is observed in A549 pulmonary adenocarcinoma cells, where its genetic silencing substantially abrogates cisplatin‐induced G2/M arrest, enhances apoptotic rates, and improves chemosensitivity [21, 22]. In Lewis lung carcinoma models, combined CHEK1‐targeted RNA interference (RNAi) and radiotherapy synergistically inhibit tumor growth while reducing CHEK1 mRNA expression [23, 24]. Emerging evidence further connects CHEK1 dysregulation to tumor microenvironment remodeling, as demonstrated in multiple myeloma where CHEK1 and its circular RNA derivative (circCHEK1‐246aa) induce chromosomal instability (CIN) and promote osteoclast differentiation, thereby accelerating bone metastasis [25]. These findings collectively establish CHEK1 as a pivotal therapeutic target in cancer progression and treatment resistance.

Functional crosstalk between the PI3K/AKT pathway and CHEK1 represents a potentially critical regulatory mechanism. PI3K/AKT activation indirectly modulates CHEK1 expression and activity through several mechanisms, including AKT‐mediated phosphorylation that enhances the stability of cell cycle checkpoint proteins, consequently influencing CHEK1‐directed DNA repair responses [26, 27]. Conversely, inhibition of PI3K/AKT signaling in lung cancer frequently correlates with reduced CHEK1 activity, suggesting cooperative interactions in maintaining tumor cell survival [28, 29]. While matrine is established as a PI3K/AKT inhibitor, its potential impact on CHEK1 regulation remains unexplored. Recent evidence indicates that matrine inhibits mTOR phosphorylation downstream of PI3K/AKT, inducing tumor cell autophagy and apoptosis [12, 30]. Given mTOR's established involvement in DDR signaling, this provides a plausible mechanistic link between matrine and potential CHEK1 modulation.

This study tests the hypothesis that matrine suppresses lung cancer progression by concurrently downregulating CHEK1 and inhibiting the PI3K/AKT pathway, leading to impaired DNA repair, G2/M checkpoint abrogation, and apoptosis. These findings may provide novel mechanistic insights into natural product‐based strategies for lung cancer therapy.

## 2 Materials and Methods

### 2.1 Network Pharmacological and Bioinformatics Analysis

Active ingredients in *Sophora flavescens* were retrieved from the TCMSP database, with oral bioavailability (OB) ≥ 30% and drug‐likeness (DL) ≥ 0.18 as screening criteria. Corresponding targets of these ingredients were extracted from TCMSP and standardized to gene symbols via UniProt. Lung cancer‐related targets were obtained from GeneCards and OMIM databases using keywords “lung cancer,” “tumor,” and “oncogene.” Common targets shared between *Sophora flavescens* and lung cancer were identified through Venn analysis. The interaction network of active ingredients and overlapping targets was visualized using Cytoscape 3.10.2. Gene Ontology (GO) functional enrichment and Kyoto Encyclopedia of Genes and Genomes (KEGG) pathway analyses were performed on the overlapping targets via Metascape, with significance set at *p < 0.05. Results were visualized using R (v4.3.1). For subsequent experimental validation, the expression and prognostic relevance of key targets in lung cancer were assessed using TIMER2.0 (for immune infiltration) and GEPIA (for differential expression and survival analysis).

### 2.2 Cell Culture

BEAS‐2B (human normal bronchial epithelium), A549 (human lung adenocarcinoma), and LLC (mouse Lewis lung carcinoma) cells were cultured separately. BEAS‐2B cells were grown in MEM, A549 cells used DMEM/F12, and LLC cells used RPMI‐1640, all supplemented with 10% FBS. Cells were maintained at 37°C in a humidified 5% CO_2_ incubator. Cells were subcultured at 80%–90% confluence using 0.25% trypsin–EDTA and washed with PBS before experiments.

### 2.3 Cell Viability Assay

A549 cells were seeded in 96‐well plates at 2 × 10^4^ cells/well in 100 μL complete medium and incubated overnight. Cells were then treated with matrine (0, 1, 2.5, 5, 10, 20, 40, 80, 160, 320 μM) for 24 h, cells treated with an equivalent volume of DMSO (vehicle, final concentration ≤ 0.1%) served as the vehicle control. A 24‐h treatment duration was selected based on preliminary time‐course experiments (6, 12, 24, and 48 h), which showed that the most prominent and consistent dose‐dependent response occurred at 24 h. Prolonged exposure (48 h) resulted in excessive cytotoxicity even at lower concentrations, making it less suitable for mechanistic evaluation. After treatment, 10 μL CCK‐8 solution was added to each well (final concentration: 10%) and plates were incubated at 37°C for 45 min. Absorbance was measured at 450 nm using a microplate reader. Experiments were performed in triplicate. The half‐maximal inhibitory concentration (IC_50_) values were calculated by nonlinear regression using a four‐parameter logistic model (GraphPad Prism 8.0).

### 2.4 Cell Wound Scratch Assay

A549 and LLC cells were seeded in 6‐well plates and grown to 90%–95% confluence. Sterile 10‐μL pipette tips were used to create linear scratches. After washing with PBS to remove debris, cells were treated with matrine (0, 10, 20, 40 μM) in serum‐free medium. Images of identical scratch regions were captured at 0 h (baseline) and 24 h posttreatment using an inverted phase‐contrast microscope (10× objective). Scratch widths were quantified using ImageJ software.

### 2.5 Clone Formation Assay

A549 and LLC cells were seeded in 6‐well plates at 500 cells/well. After 24 h, cells were treated with matrine (0, 10, 20, 40 μM) for 24 h. The medium was then replaced with drug‐free complete medium and cultured for 7–10 days. Colonies were fixed with 4% paraformaldehyde, stained with 0.5% crystal violet (w/v), and imaged. Colonies containing > 50 cells were counted manually.

### 2.6 Cell Invasion Assay

The inhibitory effect of matrine on tumor cell invasion was evaluated using a Transwell chamber assay. Briefly, A549 and LLC cells were treated with various concentrations of matrine (0, 10, 20, and 40 μM) for 24 h. Subsequently, the pre‐treated cells were harvested and seeded into the upper chamber of a Matrigel‐coated insert, suspended in serum‐free medium. Complete medium containing 10% FBS was added to the lower chamber as a chemoattractant. After 24 h of incubation, the non‐invading cells on the upper surface of the membrane were carefully removed. The cells that had invaded through the Matrigel to the lower surface were fixed with 4% paraformaldehyde, stained with 0.1% crystal violet, and photographed. The number of invaded cells was quantified by counting in five random fields per well under an inverted microscope. Each experiment was performed in triplicate and repeated at least three times independently.

### 2.7 Flow Cytometry

A549 and LLC cells were seeded in 6‐well plates (2 × 10^5^ cells/well) and treated with matrine (0, 10, 20, 40 μM) for 24 h. For apoptosis, cells were harvested with trypsin, washed with PBS, and stained with Annexin V‐FITC/PI in binding buffer for 15 min (dark). For cell cycle, cells were fixed in 70% ethanol (−20°C, 2 h), and stained with PI (50 μg/mL). Samples were analyzed using a flow cytometer (488 nm excitation). Apoptotic populations (Annexin V^+^/PI^−^: early apoptosis; Annexin V^+^/PI^+^: late apoptosis) and cell cycle phases (G0/G1, S, G2/M) were quantified with FlowJo v10.8 software. A minimum of 10 000 events were recorded per sample.

### 2.8 Cell Transfection

All siRNAs were synthesized by Genepharma (Shanghai, China). The siRNA sequences were as follows: for human CHEK1, sense 5′‐CCCUCAUACAUUGAUAAAUTT‐3′ and antisense 5′‐AUUUAUCAAUGUAUGAGGGTT‐3′; for mouse Chek1, sense 5′‐GUGGGACUUUACCUUAUGUTT‐3′ and antisense 5′‐ACAUAAGGUAAAGUCCCACTT‐3′; for human PI3K (p110α), sense 5′‐GGCUAAAGAAAGCCUUUAUTT‐3′ and antisense 5′‐AUAAAGGCUUUCUUUAGCCTT‐3′; for mouse PI3K (p110α), sense 5′‐GAGCGGUACAGCAAAGAAUTT‐3′ and antisense 5′‐AUUCUUUGCUGUACCGCUCTT‐3′. A scrambled non‐targeting siRNA (sense 5′‐UUCUCCGAACGUGUCACGUTT‐3′, antisense 5′‐ACGUGACACGUUCGGAGAATT‐3′) was used as negative control. siRNAs were used at a final concentration of 50 nM. Transfection was performed using Lipofectamine 3000 (Invitrogen, catalog number L3000015) at a 1:2 (w/w) siRNA:lipofectamine ratio in Opti‐MEM reduced‐serum medium (Gibco, catalog number 31985070). Cells at 60%–70% confluence were incubated with the transfection complexes for 6 h in antibiotic‐free medium, after which the medium was replaced with fresh complete medium. For experiments combining transfection with matrine treatment, matrine was added 24 h after the start of transfection (18 h after medium replacement). Cells were harvested for downstream assays 48 h after the start of transfection (24 h after matrine addition, where applicable).

### 2.9 Western Blotting

A549 and LLC cells were seeded in 6‐well plates (2 × 10^5^ cells/well) and treated with matrine (0, 10, 20, 40 μM) for 24 h. Cells were lysed in RIPA buffer containing protease/phosphatase inhibitors. Lysates (30 μg protein) were separated by 10% SDS‐PAGE and transferred to PVDF membranes. After blocking with 5% BSA (1 h, RT), membranes were incubated overnight at 4°C with primary antibodies against: CHEK1, PI3K, p‐PI3K, AKT, p‐AKT, MMP9, MMP2, Vimentin, E‐cadherin, γH2AX, Bax, Bcl‐2, Cleaved Caspase‐3, Cyclin B1, CDK1 and p‐CDC2 (1:2000 dilution). β‐Actin (1:5000) served as a loading control. After TBST washes 3 times, membranes were incubated with HRP‐conjugated secondary antibodies (1:5000, 1 h, RT). Protein bands were visualized using ECL substrate (Bio‐Rad) and quantified by ImageLab software.

### 2.10 Immunofluorescent Staining

A549 and LLC cells were seeded on coverslips in 24‐well plates (8 × 10^4^ cells/well). After 24 h matrine treatment (0, 10, 20, 40 μM), cells were fixed with 4% paraformaldehyde (15 min), permeabilized with 0.5% Triton X‐100 (10 min), and blocked with 5% BSA (1 h). Primary antibodies against CHEK1 (1:200) and p‐PI3K (1:200) were applied overnight at 4°C. After PBS washes, cells were incubated with Alexa Fluor 488/594 secondary antibodies (1:500). Nuclei were counterstained with DAPI (5 μg/mL, 5 min). Images were acquired using a confocal microscope.

### 2.11 Animals and Experimental Groups

32 male C57BL/6 mice (6 weeks old, 18–22 g) were obtained from the Laboratory Animal Center of Zhejiang University (SCXK[Zhe]2022‐0003) and housed under specific pathogen‐free conditions at 22°C ± 1°C with 55% ± 5% humidity and a 12‐h light/dark cycle, with free access to autoclaved food and water. Following a 7‐day acclimatization period LLC cells (5 × 10^5^ cells in 100 μL PBS) were subcutaneously inoculated into the right flank. When tumors reached approximately 100 mm^3^ (Day 7 postinoculation), mice were randomized into four treatment groups (*n* = 8/group): control (saline), low‐dose (10 mg/kg matrine), medium‐dose (20 mg/kg matrine), and high‐dose (40 mg/kg matrine). Intratumoral injections (50 μL volume) were administered every 48 h for 14 days.

Tumor dimensions were measured daily using digital calipers, with volumes calculated as (Length × Width^2^)/2. On Day 21, mice were euthanized via CO_2_ asphyxiation followed by cervical dislocation. For downstream analysis, tumor samples were either snap‐frozen in liquid nitrogen for Western blotting or fixed in 4% paraformaldehyde for 24 h followed by paraffin embedding and sectioning (4 μm) for immunohistochemical analysis. All procedures complied with NIH guidelines and were approved by the Institutional Animal Ethics Committee of Zhejiang University (Protocol ZJU20250860).

A toxicity study was conducted using 24 mice randomly divided into four groups (*n* = 6 per group). All animals received a single intraperitoneal injection of matrine at a dose of 40 mg/kg (The selected doses (10, 20, 40 mg/kg) were based on a pilot dose‐finding study, relevant published reports demonstrating antitumor efficacy of matrine within this dose range [9, 31], and the in vitro IC50 values (approximately 30–44 μM), aiming to achieve local drug exposure comparable to or higher than the effective in vitro concentrations while avoiding systemic toxicity). Blood samples and major organs (including liver, kidney, heart, lung, and spleen) were collected at predetermined time points: immediately before administration (Day 0), and on Day 1, Day 5, and Day 10 postinjection. Serum biochemical parameters were analyzed to assess organ function and potential damage. For histopathological examination, the collected tissues were fixed in 10% neutral buffered formalin, embedded in paraffin, sectioned, and stained with hematoxylin and eosin (H&E) for microscopic evaluation.

### 2.12 Hematoxylin–Eosin (HE) Staining

Paraffin‐embedded tumor sections (4 μm) were deparaffinized in xylene and rehydrated through graded ethanol series. Nuclei were stained with Mayer's hematoxylin for 5 min, followed by differentiation in 1% acid ethanol and bluing in 0.2% ammonia water. Cytoplasmic counterstaining was performed with 0.5% eosin for 3 min. Sections were dehydrated through ethanol, cleared in xylene, and mounted with neutral balsam.

### 2.13 Immunohistochemical (IHC) Staining

Deparaffinized tumor sections (4 μm) underwent antigen retrieval in citrate buffer (pH 6.0, 95°C, 20 min). Endogenous peroxidase was blocked with 3% H_2_O_2_ (15 min). After blocking with 5% BSA, sections were incubated overnight at 4°C with primary antibodies: anti‐CHEK1 (1:200), anti‐Ki67 (1:100), anti‐Bax (1:200), and anti‐p‐PI3K (1:100). HRP‐conjugated secondary antibodies (1:500) were applied (1 h, RT), followed by DAB development and hematoxylin counterstaining.

### 2.14 Biochemical Assays

Blood collected via cardiac puncture at euthanasia was centrifuged (3000 × *g*, 15 min, 4°C) to obtain serum. AST, ALT, BUN, and CREA were quantified using a FUJI DRI‐CHEM NX700i dry chemistry analyzer (Fujifilm). Serum samples (10 μL) were applied directly to dedicated dry slides. Reflectance photometry measurements were performed according to manufacturer protocols. Results were calculated automatically against pre‐calibrated standards and expressed as mean ± SD (*n* = 8/group). All experiments were performed with at least three independent biological replicates. For in vitro assays, each biological replicate included 3 technical replicates. In all figures, ‘*n*’ denotes the number of independent biological replicates.

### 2.15 Statistical Analysis

All experiments were performed at least three independent times, unless otherwise specified. Quantitative data are presented as mean ± standard deviation (SD). Normality of data distributions was assessed using the Shapiro–Wilk test. For comparisons between two groups, a two‐tailed unpaired Student's *t*‐test was used. For comparisons among three or more groups, one‐way analysis of variance (ANOVA) was performed, followed by Tukey's post hoc test for multiple comparisons. A *p* value of less than 0.05 was considered statistically significant. All statistical analyses were carried out using GraphPad Prism software (version 8.0). In all figures, significance levels are denoted as: *p < 0.05, **p < 0.01, ***p < 0.001.

## 3 Results

### 3.1 Network Pharmacology of Matrine in the Treatment of Lung Cancer and Key Target Analysis

**FIGURE 1:**
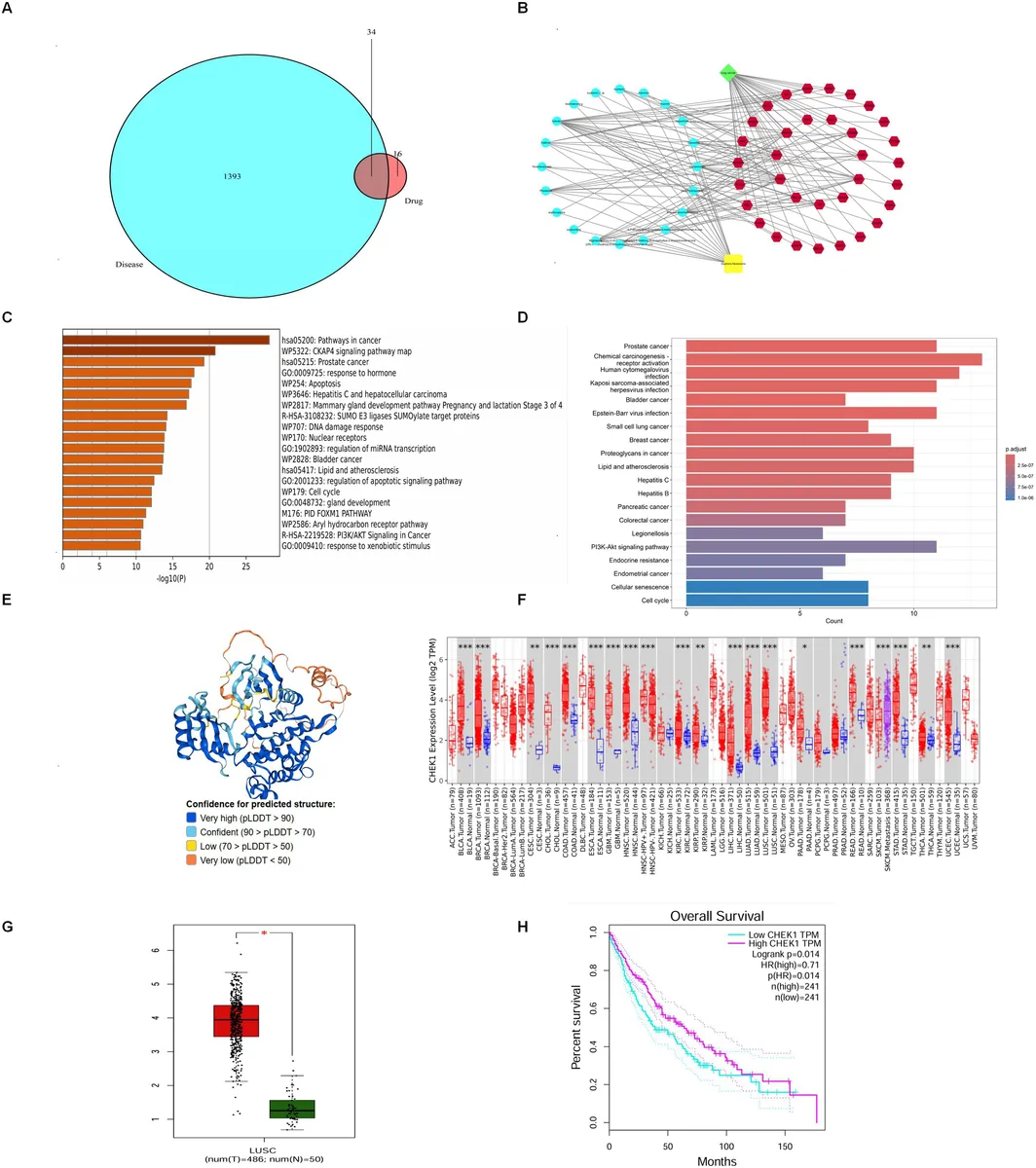
Integrated network pharmacology and CHEK1 expression analysis of *Sophora flavescens* in lung squamous cell carcinoma (LUSC). (A) Venn diagram of 34 shared targets between bioactive compounds of *S. flavescens* and LUSC. (B) Compound‐target‐disease interaction network: *S. flavescens* (yellow), compounds (light blue), targets (red). (C, D) Top 20 enriched KEGG pathways and GO biological processes, respectively (sorted by *p*‐value). (E) Tertiary structure of human CHEK1 protein (*Source:* The Human Protein Atlas; ID: ENSG00000149554). (F, G) CHEK1 mRNA overexpression across multiple cancers (TIMER2; ***p < 0.001). (H) Validation of CHEK1 upregulation in TCGA cohorts: 2.3‐fold in LUAD and 3.1‐fold in LUSC (GEPIA; FDR < 0.01). Bioactive components were sourced from TCMSP; LUSC targets from GeneCards and OMIM. Networks were constructed using Cytoscape 3.10.2; functional enrichment performed in R (*p < 0.05).

Through screening in the TCMSP database, 45 bioactive components of *Sophora flavescens* were identified, including matrine and flavonoid compounds such as luteolin. GeneCards and OMIM databases yielded 1427 nonredundant targets associated with lung squamous cell carcinoma (LUSC). Intersection analysis via an online Venn tool revealed 34 shared targets (Figure 1A), linked to 50 component‐specific targets (IL6, PCNA) and 20 active ingredients. A compound‐target‐disease network was constructed using Cytoscape 3.10.2 (Figure 1B), where *Sophora flavescens* is represented in yellow, phytochemicals in light blue, and LUSC‐shared targets in red.

GO and KEGG enrichment analyses of the shared targets were performed in R. The top 20 enriched terms (ranked by *p*‐value) are visualized in Figure 1C,D. KEGG analysis demonstrated significant enrichment in the PI3K/AKT signaling pathway, infections, multiple cancers, and endocrine resistance (Figure 1C). GO biological processes included apoptosis, DDR, and hormone‐mediated signaling pathways (Figure 1D). These results underscore the multitarget and multipathway mechanisms underlying *Sophora flavescens* 's anticancer effects, highlighting its potential role in modulating critical oncogenic processes.

### 3.2 Bioinformatics Analysis Reveals Upregulation of chek1 Expression in Lung Cancer

Based on network pharmacology findings, matrine‐a bioactive alkaloid in *Sophora flavescens* ‐was prioritized for further investigation. Among the shared targets between *Sophora flavescens* and lung cancer, CHEK1 was identified as a critical hub gene with established roles in cancer pathogenesis (Figure 1B). The tertiary structure of human CHEK1 protein was resolved using The Human Protein Atlas (Figure 1E). Transcriptomic analysis via TIMER 2.0 and GEPIA databases revealed significant overexpression of CHEK1 in lung cancer tissues compared to normal controls (***p < 0.001), with consistent upregulation observed across multiple datasets (Figure 1F–H). Specifically, CHEK1 expression in LUAD (lung adenocarcinoma) and LUSC cohorts showed 2.3‐fold and 3.1‐fold increases, respectively (GEPIA; FDR < 0.01). These results implicate CHEK1 as a druggable oncogenic target in lung cancer and suggest that matrine exerts antitumor effects through CHEK1 modulation. Experimental validation of this mechanism follows.

**FIGURE 2:**
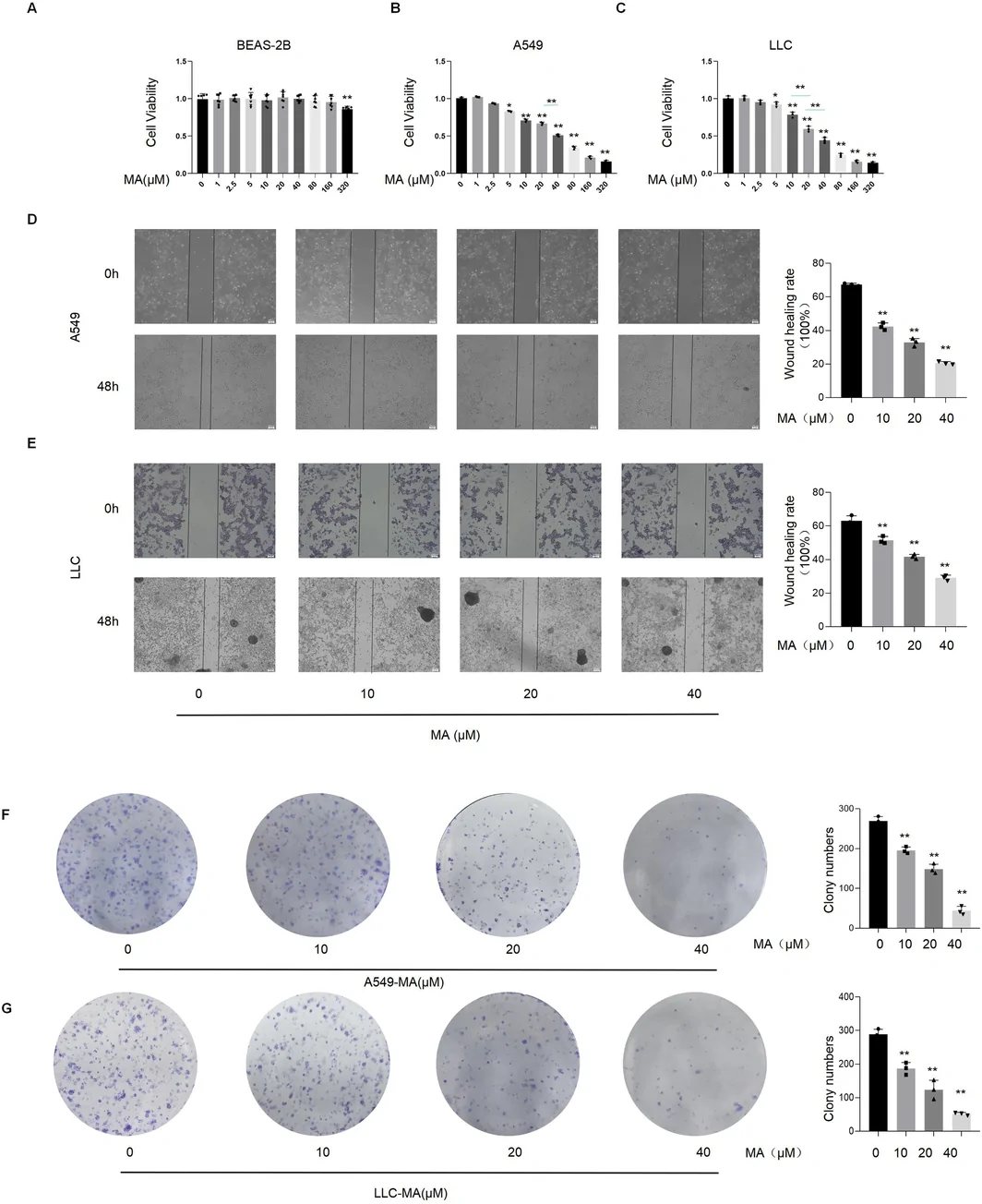
Matrine exerts selective cytotoxicity, inhibits migration, and suppresses clonogenicity in lung cancer cells. (A) Viability of normal lung epithelial cells (BEAS‐2B) treated with matrine (0–320 μM, 24 h; CCK‐8 assay) (*n* = 3). (B, C) Dose‐dependent decrease in viability of A549 (B) and LLC (C) cells after 24 h matrine treatment (*n* = 3). (D, E) Scratch wound healing assays showing migration inhibition in A549 (D) and LLC (E) cells treated with matrine (0–40 μM, 24 h); representative images (left) and quantifications (right). Scale bars: 200 μm (*n* = 3). (F, G) Colony formation assays of A549 (F) and LLC (G) cells treated with matrine (0–40 μM, 24 h) and cultured for 7 days in drug‐free medium. DMSO was used as vehicle control. Values are expressed as mean ± SD (*n* = 3). *p < 0.05, **p < 0.01 vs. control group.

Among the 45 bioactive components of *Sophora flavescens* , matrine—the most abundant alkaloid with known antitumor activity—was selected for further investigation. Among the 34 shared targets, CHEK1 was prioritized as a hub gene with the highest connectivity in the compound‐target network and showed significant overexpression in lung cancer tissues (≥ 2.3‐fold, FDR < 0.01).

### 3.3 Matrine Exhibits Selective Cytotoxicity Against Lung Cancer Cells With Minimal Impact on Normal Pulmonary Cells

As demonstrated in Figure 2A, matrine treatment induced negligible toxicity in normal lung cells (BEAS‐2B) even at 320 μM, maintaining 86% cell viability relative to untreated controls. In contrast, significant dose‐dependent cytotoxic effects were observed in malignant cells. A549 cells showed moderate sensitivity to matrine, with 5 μM eliciting marginal anti‐proliferative activity (*p < 0.05). Cytotoxicity increased progressively at higher concentrations, reaching 52% viability reduction (vs. control, **p < 0.01) at 40 μM (Figure 2B). Notably LLC cells exhibited enhanced susceptibility, where 40 μM matrine reduced viability to 44% of control levels (**p < 0.01; Figure 2C) (The raw data for Figure 2B,C are provided in Table S1). These results demonstrate matrine's selective anti‐tumor efficacy and suggest a therapeutic window between cancerous and normal pulmonary cells. The half‐maximal inhibitory concentration (IC_50_) values of matrine were calculated from the dose–response curves using nonlinear regression analysis (four‐parameter logistic model). The IC_50_ values were 44.1 μM (95% CI: 38.7–51.0) for A549 cells and 32.3 μM (95% CI: 26.9–37.2) for LLC cells.

### 3.4 Matrine Inhibits Cell Migration in A549 and LLC Cells

As shown in Figure 2D,E, matrine treatment significantly suppressed the migratory capacity of A549 and LLC cells in a dose‐dependent manner. Representative images of scratch wounds at 0 and 24 h posttreatment demonstrated markedly delayed wound closure in both cell lines following matrine exposure. Quantitative analysis revealed that 40 μM matrine reduced wound closure rates to 20.57% ± 0.53% (A549) and 29.1% ± 1.57% (LLC) relative to untreated controls (set as 100%, **p < 0.01). These findings confirm matrine's potent anti‐metastatic activity through targeted inhibition of lung cancer cell motility.

### 3.5 Matrine Inhibits Clonogenic Survival of A549 and LLC Cells

Matrine treatment significantly suppressed the clonogenic survival of both A549 and LLC cells in a dose‐dependent manner. As illustrated in Figure 2F,G, exposure to increasing concentrations of matrine (10, 20, and 40 μM) resulted in a progressive reduction in both the number and size of the colonies formed compared to the untreated control (0 μM). Quantitative analysis demonstrated that matrine at 40 μM almost completely abolished colony formation, inhibiting the clonogenic ability by over 80% in both cell lines. The differences between all treatment groups and the control were statistically significant (*p* < 0.01). These results indicate that matrine potently inhibits the proliferative capacity and long‐term survival of lung cancer cells.

### 3.6 Matrine Exerts Multimodal Antitumor Effects by Suppressing Invasion, Inducing Apoptosis, and Arresting Cell Cycle Progression

**FIGURE 3:**
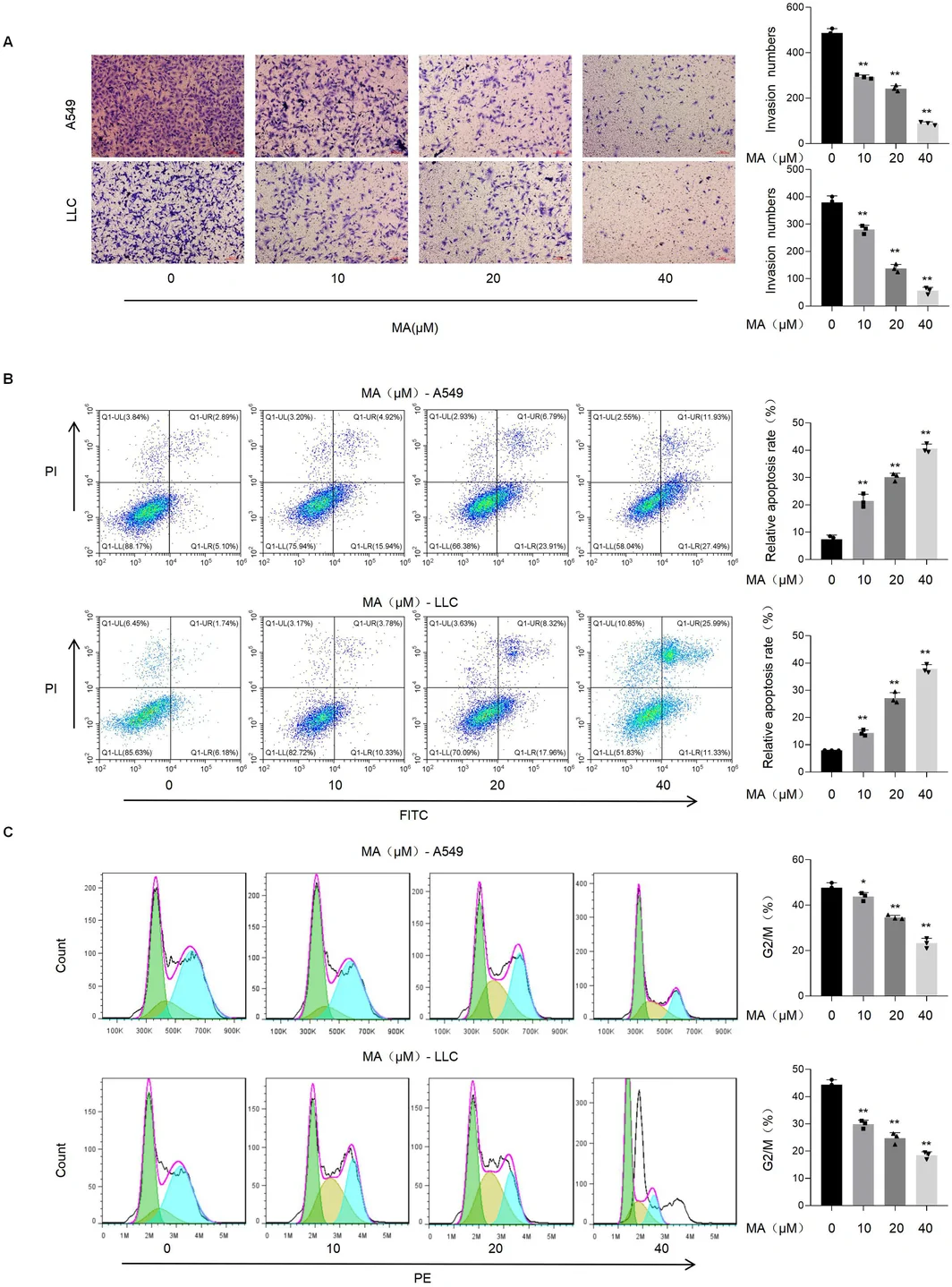
Matrine potently inhibits invasion, induces apoptosis, and arrests cell cycle in lung cancer cells. (A) Matrigel invasion assays of A549 and LLC cells treated with matrine (0–40 μM, 24 h) (*n* = 3). (B) Flow cytometric apoptosis analysis (Annexin V/PI staining) in A549 and LLC (*n* = 3). (C) Cell cycle distribution in A549 and LLC after 24 h treatment. Values are expressed as mean ± SD (*n* = 3). *p < 0.05, **p < 0.01 vs. control group.

Matrine treatment significantly inhibited the invasive capacity of both A549 and LLC cells in a concentration‐dependent manner, as assessed by the Transwell chamber assay. Compared to the untreated control (0 μM), the number of invaded cells was markedly reduced across all treatment groups. Quantitative analysis revealed that matrine at concentrations of 10, 20, and 40 μM reduced invasion by approximately 39%, 49%, and 82% in A549 cells, and by 26%, 53%, and 84% in LLC cells, respectively. The differences between the treatment groups and the control were statistically significant (*p* < 0.01 for 10, 20, and 40 μM). These results clearly demonstrate that matrine possesses a potent anti‐invasive effect on both tested lung carcinoma cell lines (Figure 3A).

As shown in Figure 3B, Flow cytometric analysis with Annexin V‐FITC/PI staining demonstrated that matrine effectively induced apoptosis in both A549 and LLC cells in a dose‐dependent manner. Following 24 h of treatment, the total apoptotic cell population (combined early and late apoptosis) increased significantly across all treatment groups compared to the untreated control (0 μM). Quantitative analysis revealed that exposure to 10, 20, and 40 μM matrine resulted in approximately 21%, 30%, and 40% apoptosis in A549 cells, and 14%, 27%, and 38% in LLC cells, respectively. The differences between the treated groups and the control were statistically significant (*p* < 0.01 for 10, 20, and 40 μM). These results clearly indicate that matrine triggers programmed cell death as one of its anti‐tumor mechanisms.

Matrine decreased the G2/M population, consistent with CHEK1 inhibition‐mediated checkpoint abrogation (Figure 3C). Matrine (40 μM) significantly decreased G2/M populations to 23.28% ± 2.5% (A549) and 18.43% ± 3.1% (LLC) (***p < 0.001). These coordinated effects on invasion, apoptosis, and cell cycle machinery underscore matrine's multimodal therapeutic potential. Consistent with CHEK1 downregulation, Western blot analysis further demonstrated that matrine decreased p‐CDC2 levels, increased Cyclin B1 expression, and did not alter total CDK1 protein levels (Figure S1).

### 3.7 Matrine Suppresses CHEK1‐PI3K/AKT Signaling Axis to Inhibit Metastasis and Promote Apoptosis in Lung Cancer

**FIGURE 4:**
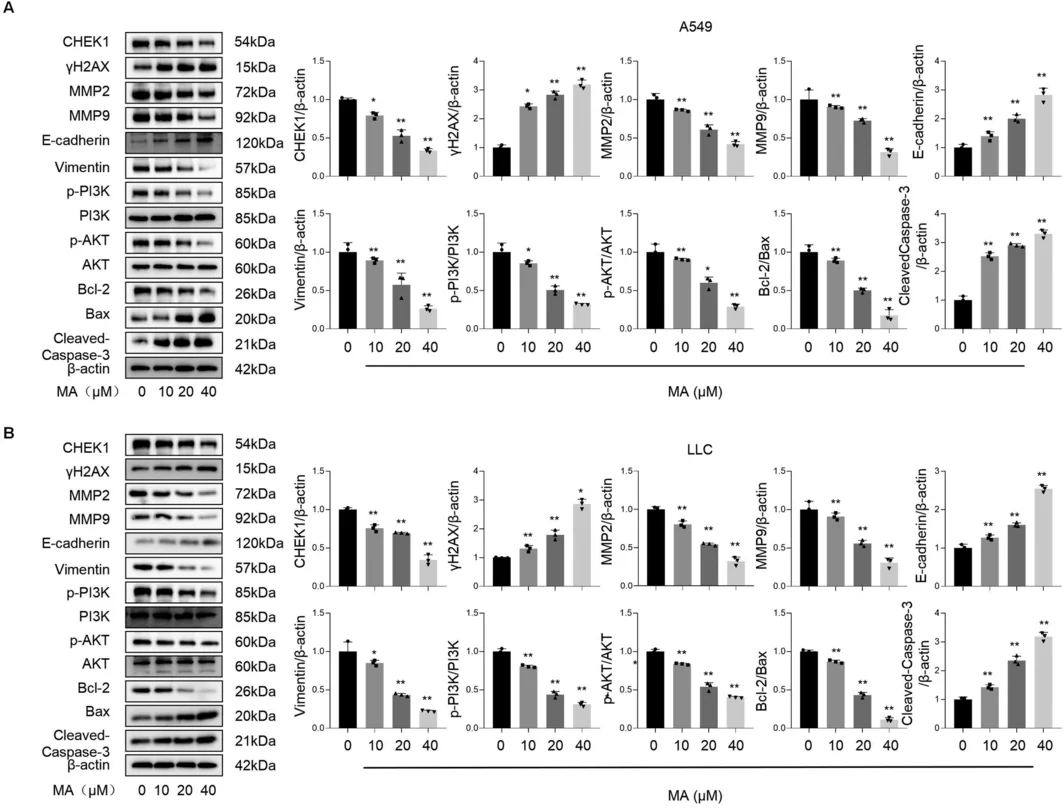
Matrine suppresses the CHEK1‐PI3K/AKT signaling axis and modulates metastasis‐ and apoptosis‐related proteins in lung cancer cells. Western blot analysis of key signaling molecules in (A) A549 and (B) LLC cells after matrine treatment, including DNA damage response (CHEK1, γH2AX), metastasis‐related factors (MMP2, MMP9, E‐cadherin, Vimentin), PI3K/AKT pathway activity (p‐PI3K, PI3K, p‐AKT, AKT), and apoptosis regulators (Bcl‐2, Bax, Cleaved Caspase‐3). Values are expressed as mean ± SD (*n* = 3). *p < 0.05, **p < 0.01 vs. control group.

As shown in Figure 4, Western blot analysis revealed that matrine treatment dose‐dependently suppressed the expression of key proteins involved in tumor progression across multiple pathways. Notably, matrine significantly downregulated the levels of cell cycle regulator (CHEK1), phospho‐PI3K, phospho‐AKT, metastasis‐associated proteins (MMP2, MMP9, Vimentin), and the anti‐apoptotic protein BCL‐2. Concurrently, matrine treatment markedly upregulated proteins associated with DNA damage (γH2AX), epithelial phenotype (E‐cadherin), and pro‐apoptotic signaling (Bax and Cleaved‐Caspase‐3). These alterations in protein expression demonstrate that matrine exerts its anti‐tumor effects by concurrently inducing DNA damage, inhibiting PI3K/AKT signaling, suppressing epithelial‐mesenchymal transition (EMT), and activating mitochondrial apoptosis.

**FIGURE 5:**
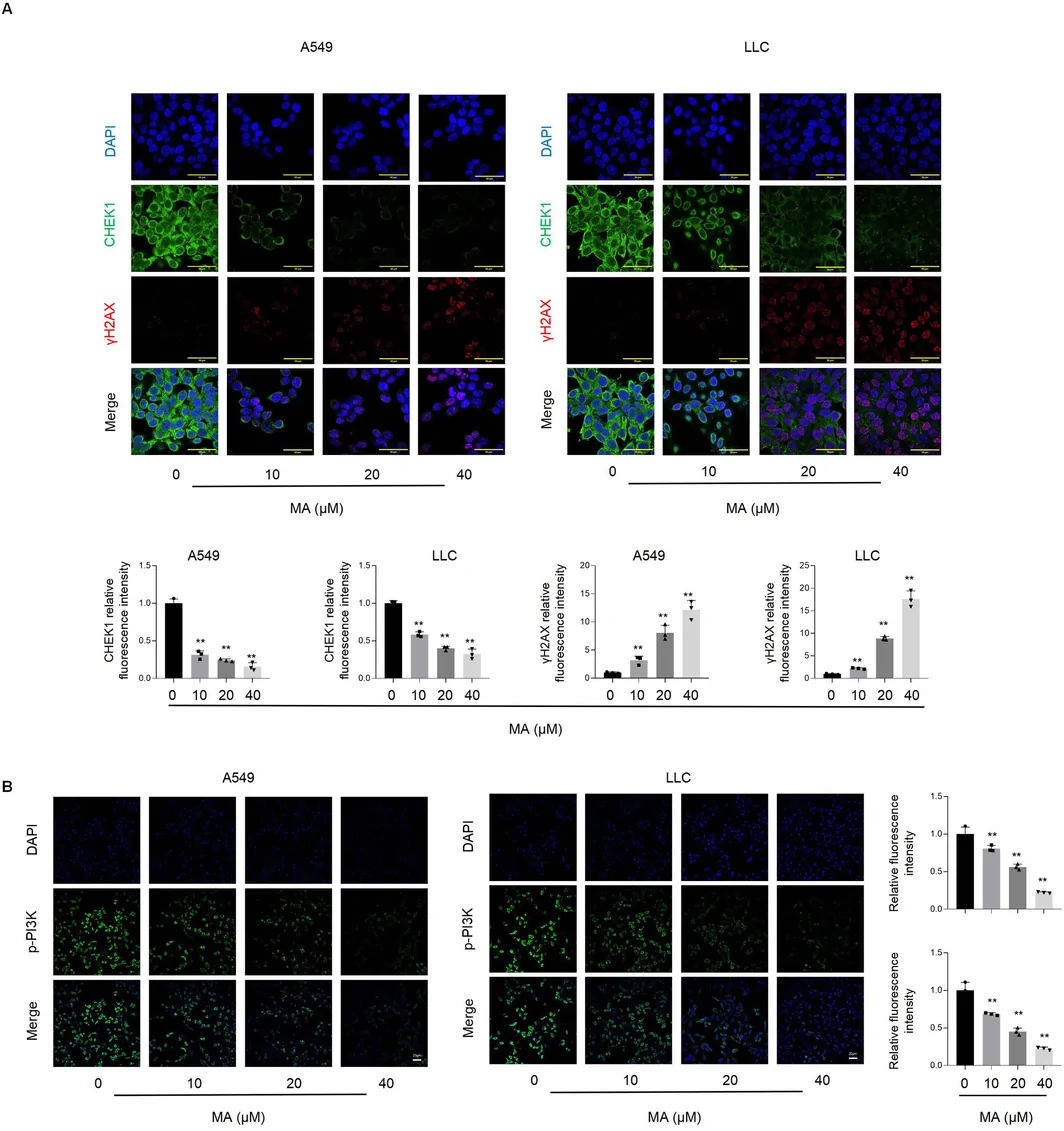
Matrine modulates the expression and subcellular localization of CHEK1, γH2AX, and p‐PI3K in lung cancer cells. (A) Immunofluorescence images for CHEK1 and γH2AX; (B) immunofluorescence images for p‐PI3K in A549 and LLC cells after matrine intervention. Quantitative analysis data are shown as mean ± SD (*n* = 3). **p < 0.01 compared with the control group.

Immunofluorescence analysis further confirmed the multi‐target effects of matrine on key signaling pathways. A dose‐dependent increase in γH2AX fluorescence intensity and nuclear foci formation was observed, indicating robust DDR activation following matrine treatment. Concurrently, matrine exposure resulted in a significant reduction in nuclear CHEK1 signal intensity, suggesting impairment of DNA damage checkpoint signaling. Additionally, both cytoplasmic and membrane‐associated p‐PI3K fluorescence intensity exhibited marked attenuation in treated cells, demonstrating effective inhibition of PI3K/AKT pathway activation. These coordinated alterations in protein localization and expression levels provide visual evidence that matrine simultaneously induces genomic instability while suppressing critical pro‐survival signaling pathways in cancer cells (Figure 5).

### 3.8 Matrine Mediates Antitumor Effects via Independent Modulation of CHEK1‐Mediated DNA Damage and PI3K/AKT Apoptotic Pathways

**FIGURE 6:**
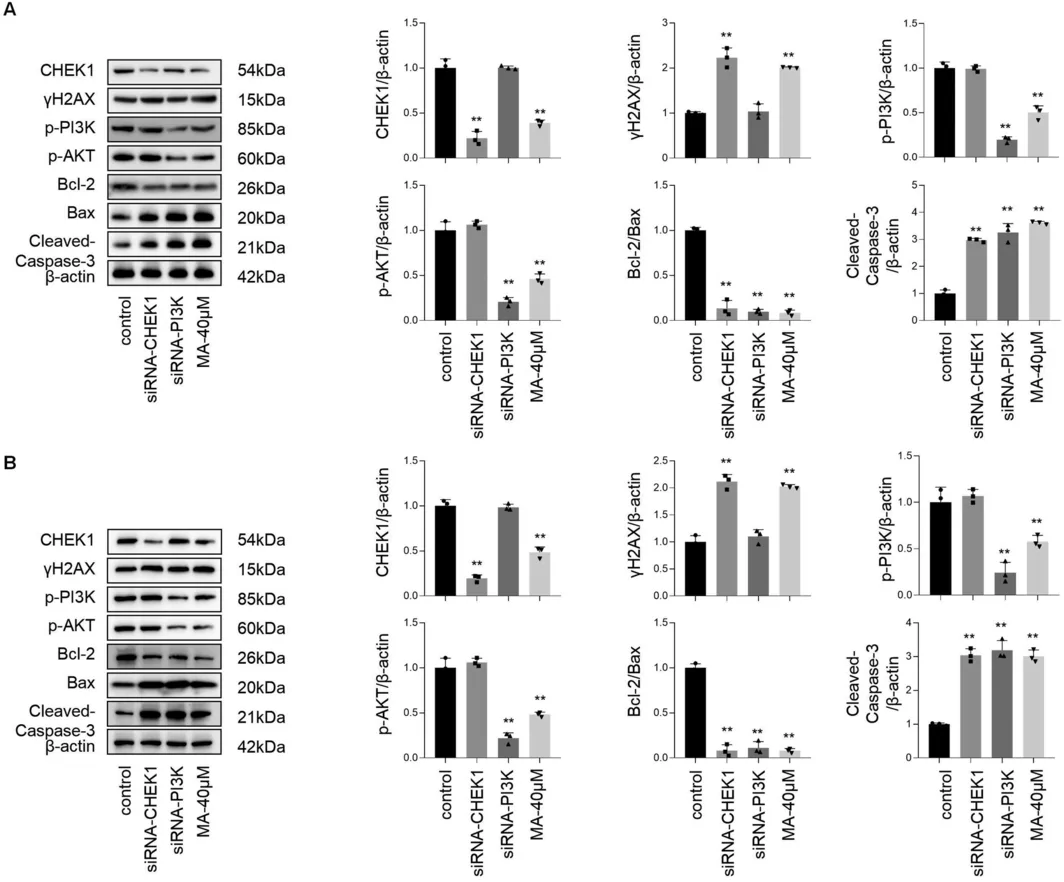
Matrine independently affects both CHEK1‐mediated DNA damage and PI3K/AKT apoptotic pathways. Western blot analysis of (A) A549 and (B) LLC cells under four treatment conditions: control, CHEK1 siRNA, PI3K siRNA, and matrine (40 μM). The results demonstrate that matrine alone simultaneously downregulates CHEK1 and inhibits PI3K/AKT signaling, phenocopying the combined effects of CHEK1 and PI3K knockdown. Values are expressed as mean ± SD (*n* = 3). **p < 0.01 vs. control group.

In Figure 6, Western blot analysis demonstrated that matrine (40 μM) significantly inhibited tumor progression in both A549 and LLC cells, as evidenced by concurrent downregulation of CHEK1, p‐PI3K, p‐AKT, and BCL‐2, along with upregulation of γH2AX, Bax, and Cleaved‐Caspase‐3. Interestingly, PI3K silencing effectively reduced p‐PI3K and p‐AKT levels and promoted apoptosis but did not alter CHEK1 or γH2AX expression. Similarly, CHEK1 knockdown induced apoptosis without affecting p‐PI3K or p‐AKT levels. In contrast, matrine treatment simultaneously suppressed both CHEK1 and PI3K/AKT pathways while strongly activating DNA damage and apoptotic markers, suggesting additive effects of action that collectively enhances its anti‐tumor efficacy.

### 3.9 Matrine Dose‐Dependently Inhibits Tumor Growth and Suppresses Proliferation in LLC Xenografts

**FIGURE 7:**
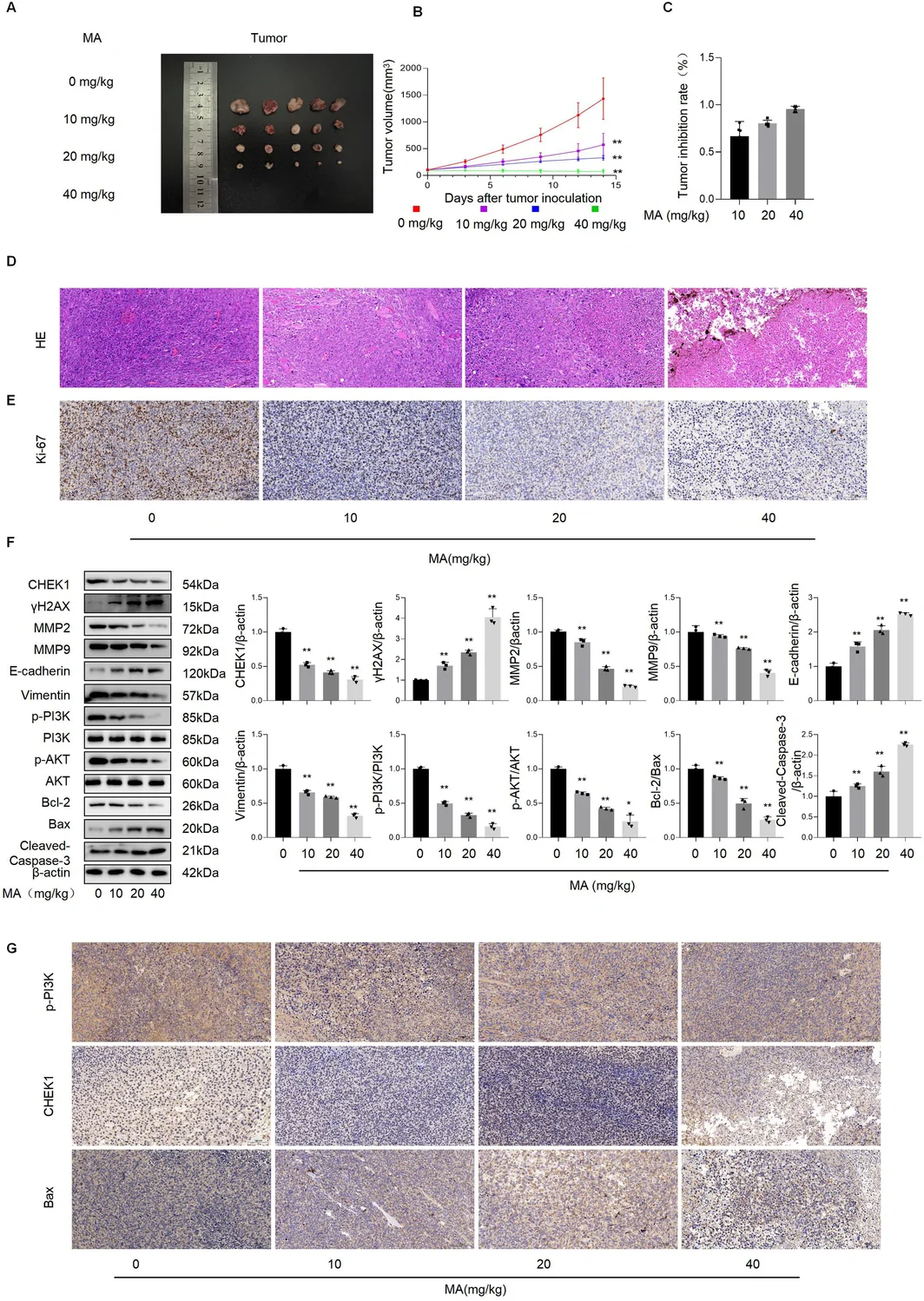
Matrine suppresses tumor growth, proliferation, and CHEK1‐PI3K/AKT signaling in vivo. (A) Representative images of excised tumors from LLC‐bearing mice after 14 days of intratumoral treatment with matrine (0, 10, 20, 40 mg/kg) (*n* = 8). (B) Time‐dependent tumor volume growth curves (*n* = 8). (C) Tumor inhibition rate across dose groups. (D, E) H&E staining and Ki67 immunohistochemistry of tumor tissues, respectively (*n* = 3). (F) Western blot analysis of key proteins in the CHEK1‐PI3K/AKT pathway from xenograft tissues (*n* = 3). (G) Representative IHC staining of CHEK1 and p‐PI3K in tumor sections. Values are expressed as mean ± SD (*n* = 3). *p < 0.05, **p < 0.01 vs. control group.

As shown in Figure 7A–C, intratumoral administration of matrine (10, 20, 40 mg/kg) significantly suppressed subcutaneous LLC tumor growth in C57BL/6 mice over 14 days. Final tumor volumes decreased dose‐dependently to 559 ± 78 mm^3^ (10 mg/kg), 331 ± 64 mm^3^ (20 mg/kg), and 72 ± 42 mm^3^ (40 mg/kg) versus 1443 ± 98 mm^3^ (control, ***p < 0.001), corresponding to inhibition rates of 66.8%, 80.3%, and 95.6%, respectively.

Histopathological analysis (Figure 7D) revealed pronounced intratumoral necrosis (≥ 70% area at 40 mg/kg) and reduced mitotic figures in matrine‐treated groups compared to hypercellular control tumors. Concordantly, Ki67 IHC demonstrated dose‐dependent reduction of proliferating cells (Figure 7E). These findings confirm matrine's potent in vivo anti‐tumor efficacy through dual suppression of tumor growth and proliferation.

### 3.10 Matrine Orchestrates Multifaceted Antitumor Mechanisms In Vivo via CHEK1‐PI3K/AKT Axis Modulation

Western blot analysis (Figure 7F) revealed significant upregulation of CHEK1, p‐PI3K, and other oncogenic markers (Vimentin, γH2AX, Bax, cleaved caspase‐3) in control tumors versus normal tissues (***p < 0.001), while matrine treatment (10–40 mg/kg) dose‐dependently suppressed these targets (≥ 2.5‐fold reduction at 40 mg/kg, **p < 0.01) and upregulated E‐cadherin (2.5‐fold). Critically, immunohistochemical staining (Figure 7G) corroborated these findings, demonstrating parallel dose‐dependent reductions in CHEK1 and p‐PI3K protein expression. This multimodal validation—spanning immunoblotting and spatial tissue profiling—confirms matrine's potent in vivo suppression of the CHEK1‐PI3K/AKT signaling hub.

### 3.11 Matrine Demonstrates Favorable Safety Profile at Antitumor Doses With no Significant Organ Toxicity

**FIGURE 8:**
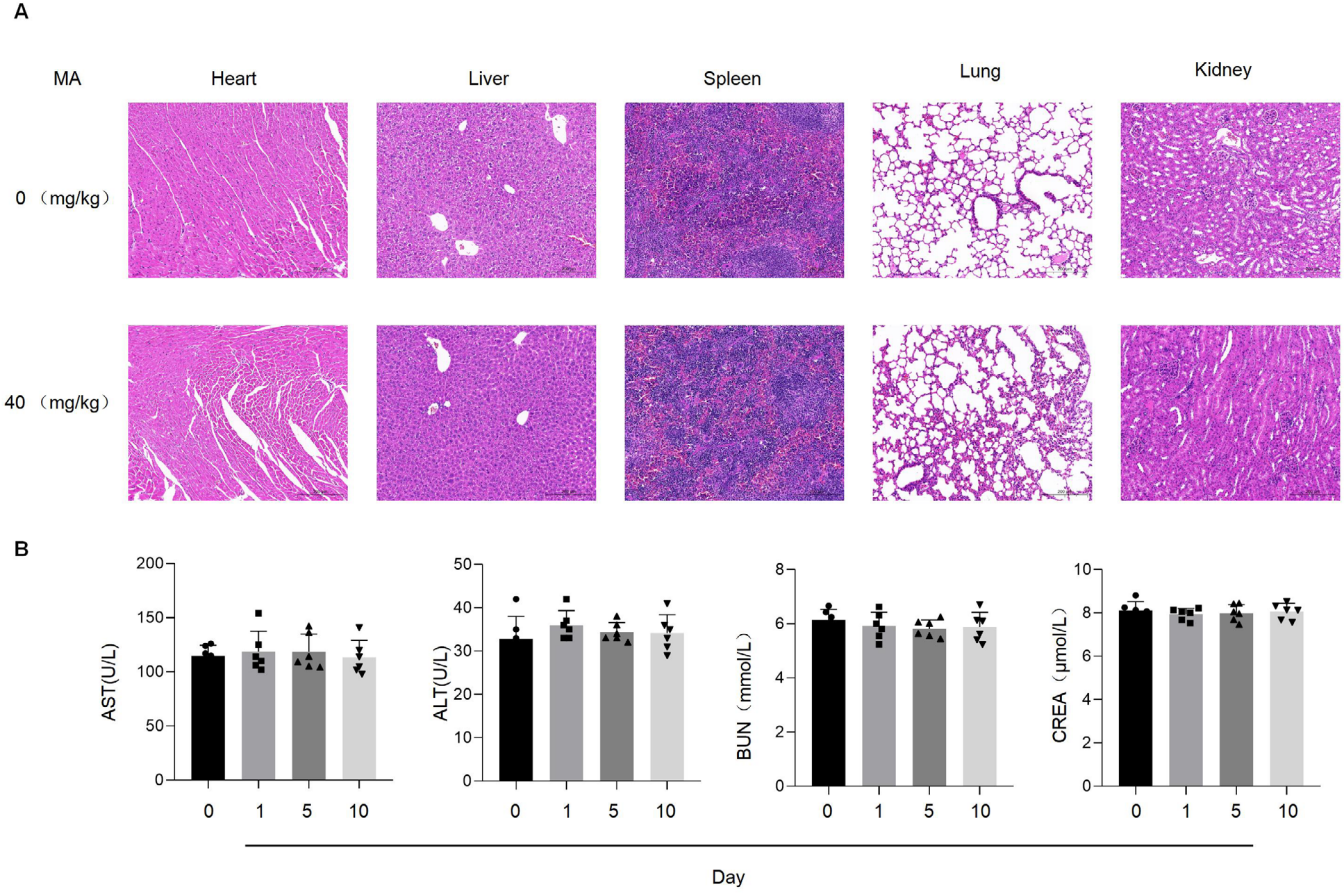
Matrine exhibits no detectable toxicity at therapeutic doses. (A) H&E staining of major organs (heart, liver, spleen, lung, kidney) from mice treated with 40 mg/kg matrine for 10 days (*n* = 3). (B) Serum biochemistry: ALT, AST, BUN, and CREA levels in control and matrine‐treated groups (40 mg/kg). Values are expressed as mean ± SD (*n* = 6).

Comprehensive toxicological assessment confirmed the biosafety of matrine at therapeutic doses. Histopathological examination of major organs (heart, liver, spleen, lung, kidney) via H&E staining revealed no significant alterations in tissue architecture, cellular integrity, or inflammatory infiltration after 10 days of 40 mg/kg matrine treatment (Figure 8A), indicating absence of acute organ damage. Concordantly, serum biochemical analysis (Figure 8B) showed levels of hepatic markers (ALT: 34.17 ± 3.5 U/L; AST: 113.3 ± 3.7 U/L) and renal parameters (BUN: 5.88 ± 2.1 mmol/L; CREA: 8.07 ± 0.13 μmol/L) within normal physiological ranges (*p < 0.05 vs. untreated controls), confirming preserved hepatic and renal function. These findings establish a therapeutic window for matrine wherein antitumor efficacy (40 mg/kg) is achieved without detectable systemic toxicity.

## 4 Discussion

This study establishes a novel mechanism involving concurrent modulation of CHEK1 and PI3K/AKT pathways. The therapeutic significance of this finding is underscored by the persistent challenges in managing advanced non‐small cell lung cancer (NSCLC), where conventional therapies frequently fail due to acquired resistance mechanisms often involving enhanced DNA repair capacity and dysregulated survival pathways [32, 33]. Against this clinical backdrop, naturally derived agents like matrine offer compelling advantages through their multi‐target engagement capabilities and favorable toxicity profiles.

Matrine, a principal alkaloid derived from *Sophora flavescens* , has been extensively documented for its multi‐target antitumor properties, encompassing the inhibition of proliferation, induction of apoptosis, cell cycle arrest, and suppression of metastasis across various cancers [34, 35, 36]. While previous studies attributed its effects to single pathways‐such as PI3K/AKT inhibition in hepatocellular carcinoma or MAPK/ERK suppression in rhabdomyosarcoma‐this study elucidates a novel dual‐pathway mechanism in lung carcinoma [37, 38, 39]. This study delineates the molecular mechanisms underpinning matrine's antitumor efficacy in lung carcinoma, integrating network pharmacology with functional validation. Our data establish that matrine concurrently modulates the CHEK1‐mediated DNA damage checkpoint and the PI3K/AKT‐driven apoptosis pathway‐resolving prior mechanistic controversies where literature attributed its effects solely to single pathways (e.g., PI3K/AKT inhibition in liver cancer or CHEK1 downregulation in thyroid cancer) [40, 41]. Critically, we demonstrate complementary: PI3K siRNA recapitulated matrine's apoptotic effects (Bax suppression, Bcl‐2 elevation) without altering γH2AX or CHEK1, while CHEK1 silencing induced DNA damage (γH2AX accumulation) and apoptosis without PI3K/AKT modulation. This dual‐pathway co‐targeting minimizes adaptive resistance‐a key limitation of monotherapies‐and positions matrine as a novel multitargeted agent. While combination experiments (siRNA plus matrine) could further quantify the additive effects of dual pathway modulation, our current data demonstrating complementary (CHEK1 knockdown does not affect PI3K/AKT, and vice versa) already establish that matrine acts simultaneously on both pathways.

CHEK1 (Checkpoint Kinase 1), a serine/threonine kinase, is a core regulator of the DNA damage checkpoint. It stabilizes replication forks during S‐phase and facilitates DNA repair by arresting the cell cycle at G2/M [42]. In malignancies, CHEK1 is frequently overexpressed to mitigate genomic instability caused by oncogenic stress. For instance, in multiple myeloma, CHEK1 overexpression drives chromosomal instability (CIN), drug resistance, and bone lesion formation [24]. Similarly, our transcriptomic analysis revealed a 3.1‐fold CHEK1 upregulation in lung cancer (Figure 2D), correlating with poor prognosis. This aligns with findings by Yang and co‐workers, who identified CHEK1 as a druggable hub in high‐risk cancers [43]. Crucially, while CHEK1's role in DNA repair is well‐established, our work newly implicates it in matrine's antitumor mechanism. Orthogonal experiments confirmed that CHEK1 silencing induced γ‐H2AX accumulation (indicating DNA damage) and apoptosis but left PI3K/AKT signaling unchanged, whereas PI3K inhibition (LY294002) suppressed apoptosis without affecting CHEK1 or γH2AX (Figure 7). This decoupling underscores matrine's unique capacity to independently co‐opt both pathways, minimizing adaptive resistance common in monotherapies.

The synergism between these pathways amplifies matrine's antitumor efficacy. Matrine treatment induced a dose‐dependent abrogation of the G2/M checkpoint in lung tumor cells, contributing to its antitumor efficacy. The reduced G2/M proportion following matrine treatment, together with decreased p‐CDC2 and sustained Cyclin B1 expression, indicates G2/M checkpoint abrogation and suggests that cells undergo mitotic catastrophe, which is consistent with the observed increase in γH2AX and apoptosis. This dual targeting explains matrine's broad activity spectrum: ≥ 2.8‐fold decrease in Bcl‐2/Bax ratio, ≥ 50% reduction in MMP2/MMP9, and 2.8‐fold E‐cadherin upregulation (Figures 4, 5, 6), collectively suppressing metastasis and activating intrinsic apoptosis. Notably, prior studies reported matrine's PI3K/AKT inhibition in liver cancer and JNK/p38 MAPK activation in lymphoma, but none described CHEK1 engagement. Our multi‐omics framework—integrating network pharmacology, transcriptomics (TIMER2.0/GEPIA), and proteomics—thus provides unprecedented mechanistic resolution.

Matrine's tumor selectivity further distinguishes it from conventional chemotherapeutics. At 40 μM, it reduced LLC viability to 30% but preserved 86% normal lung cell viability at 320 μM (Figure 3). This selectivity arises from exploiting oncogenic vulnerabilities (e.g., CHEK1 overexpression in tumors) while sparing normal tissues. By contrast, cisplatin and doxorubicin cause nephrotoxicity and cardiotoxicity [44, 45], whereas matrine induced no histopathological damage in vital organs and maintained serum ALT/AST/BUN/CREA within physiological ranges (Figure 8). Such safety is partly attributable to its natural product specificity and minimal impact on nonmalignant DNA repair (evidenced by unaltered γH2AX in normal cells posttreatment).

Despite these advances, our study has limitations. We acknowledge that the intratumoral injection used here has limited clinical applicability. However, emerging local and systemic delivery strategies, including inhalable nanoformulations and nanoparticle‐mediated targeting, may enable translational development of matrine for lung cancer therapy. Future studies using clinically relevant routes are warranted. While network pharmacology predicted direct matrine‐CHEK1 binding, molecular docking or surface plasmon resonance (SPR) validation is needed. Furthermore, matrine's immunomodulatory roles—suggested by its suppression of TNF‐α and IL‐6 in other models [46]‐remain unexplored in LUSC. Our primary in vivo anti‐tumor data were obtained from immunocompetent C57BL/6 LLC syngeneic models. Given the known immunomodulatory properties of matrine reported in previous literature, the robust tumor suppression observed in this model is likely a combined outcome of direct tumor cell inhibition and immune microenvironment regulation. To dissect the tumor‐intrinsic activity of matrine, we supplemented LLC xenograft experiments in immunodeficient nude mice. Although matrine still slowed tumor growth and suppressed the CHEK1‐PI3K/AKT pathway in nude mice, the degree of tumor shrinkage was markedly attenuated compared with the syngeneic model. This difference suggests that matrine‐mediated immune regulation likely contributes to its maximal anti‐tumor effect in immunocompetent hosts. However, we have not conducted flow cytometry analysis of tumor‐infiltrating immune cells to systematically characterize immune alterations, so we cannot fully delineate the exact immune subsets involved. Follow‐up research focusing on tumor immune microenvironment profiling will be carried out to clarify the immune‐mediated anti‐tumor effects of matrine. At the same time, the body weight data were not recorded in the original C57BL/6 therapeutic study. Nonetheless, the weight stability observed in the supplementary nude mouse experiment, combined with the histopathological and biochemical evidence from the acute toxicity study, supports the biosafety of matrine at the doses used. Future studies should include systematic body weight monitoring to further confirm long‐term tolerability (Figure S2). We also acknowledge that while our combination Western blot data suggest additive effects, quantitative functional assays would be required to formally establish pathway independence. We have therefore moderated our conclusions, referring to the two pathways as being additively or complementarily targeted by matrine, rather than strictly nonredundant (Figure S3). We also acknowledge that a standard chemotherapeutic agent (e.g., cisplatin) was not included as a positive control to benchmark matrine's antiproliferative activity. However, as our primary objective was to elucidate the mechanistic pathway rather than to compare potency against existing drugs, we focused on vehicle‐controlled comparisons. This represents a limitation of the present study.

Accordingly, we describe the two pathways as nonredundant rather than strictly orthogonal.

## 5 Conclusion

In summary, this study identifies matrine as a natural agent that simultaneously downregulates CHEK1 and PI3K/AKT signaling in lung cancer. The concurrent modulation of these two pathways, which function in a complementary rather than vertically connected manner, likely contributes to matrine's antitumor efficacy by impairing DNA damage repair and disabling survival signals. However, given the multi‐target nature of natural products and the complexity of in vivo systems, we acknowledge that additional pathways—including potential immunomodulatory effects—may also participate in the overall therapeutic response. The bioinformatics‐to‐experimental validation approach employed here provides a useful framework for dissecting the polypharmacological actions of natural compounds. These findings offer a mechanistic basis for further exploration of matrine in lung cancer therapy, while recognizing that the full spectrum of its in vivo targets remains to be elucidated.

## Author Contributions

**Jianghao Yu:** investigation, writing – review and editing. **Siyang Chen:** validation. **Tingting Hou:** software. **Xianwu Weng:** methodology. **Yakun Liu:** visualization. **Junchao Huang:** investigation. **Zeshan Yang:** data curation. **Jinyu Bao:** supervision. **Changshuai Zhou:** resources. **Wanqing Lv:** resources. **Qingzhe Wu:** writing – review and editing. **Yun Xu:** writing – review and editing. **Jinshan Zhou:** writing – original draft.

## Funding

This work was supported by grants from the National Natural Science Foundation of China (No. 82100029). This research was supported by the Huadong Medicine Joint Fund of Zhejiang Provincial Natural Science Foundation of China under Grant No. LHDMZ26H160002 and LHDMZ25H290002, and Science and Technology program of Jinhua Science and Technology Bureau (Grant No. 2024‐3‐129).

## Disclosure

We confirm that no generative AI tools were used in the preparation of this manuscript.

## Conflicts of Interest

The authors declare no conflicts of interest.

## Supporting information


**Figure S1:** Matrine modulates G2/M checkpoint regulators in A549 and LLC cells. (A) A549 cells were treated with matrine (0, 10, 20, 40 μM) for 24 h. Protein expression of Cyclin B1, CDK1, and p‐CDC2 was analyzed by Western blot. β‐Actin served as loading control. Data are representative of three independent experiments.

**Figure S2:** Supplementary in vivo data of matrine against LLC xenograft tumors in nude mice. (A) Images of isolated tumors at Day 10 (*n* = 5); (B) time‐dependent tumor volume growth curves (*n* = 5); (C) body weight changes of tumor‐bearing mice over 10 days of treatment (*n* = 5); (D) Western blot detection of target proteins in tumor tissues (*n* = 5). Data are mean ± SEM. *p < 0.05, **p < 0.01 vs. control.

**Figure S3:** Combination of CHEK1 or PI3K siRNA with matrine additively modulates apoptosis‐related protein expression in A549 cells. (A) A549 cells were transfected with CHEK1 siRNA, PI3K siRNA, or control siRNA, followed by matrine treatment (40 μM). Protein expression of CHEK1, γH2AX, p‐PI3K, p‐AKT, Bcl‐2 and Bax, was analyzed by Western blot. β‐Actin served as loading control.

**Table S1:** Raw data of the CCK‐8 assays shown in Figure 2B,C. The data are presented from three independent experiments.

## Data Availability

The data generated in the present study may be requested from the corresponding author.
